# Methods for invasive species control are transferable across invaded areas

**DOI:** 10.1371/journal.pone.0187265

**Published:** 2017-11-03

**Authors:** Takashi Haramura, Michael R. Crossland, Hirohiko Takeuchi, Richard Shine

**Affiliations:** 1 The Hakubi Center for Advanced Research, Kyoto University, Sakyo, Kyoto, Japan; 2 Field Science Education and Research Center, Kyoto University, Shirahama, Wakayama, Japan; 3 School of Life and Environmental Sciences, The University of Sydney, New South Wales, Australia; 4 College of Bioresource Sciences, Nihon University, 1866 Kameino Fujisawa, Kanagawa, Japan; Universitat Zurich, SWITZERLAND

## Abstract

Cane Toads (*Rhinella marina*) are invasive pests in many parts of the world, including the Japanese island of Ishigaki. Extensive research in Australia has identified promising new methods for control, but also has shown that toads exhibit geographic variation in many traits (suggesting that methods developed in one location may not work in another). Can the approaches developed in Australia play a useful role for controlling this invasive species in Japan? Our experimental trials on Ishigaki Island suggest that these new methods can be successfully applied to Japan. First, Cane Toad embryos exposed to chemical cues of conspecific tadpoles exhibited a reduction in viability (subsequent growth and development). This response appears to be species-specific, with native frog embryos not being affected by exposure to cues from toad tadpoles, and Cane Toad embryos not being affected by exposure to cues from native frog tadpoles. Second, Cane Toad tadpoles were attracted to traps containing water from conspecific eggs, and toxin from adult conspecifics. Third, adult Cane Toads were attracted to acoustic cues of calling males, with sex differences in rates of attraction to specific versions of a synthetic call (males were attracted to choruses whereas females were attracted to low-frequency calls). Our results suggest that the methods developed by Australian researchers are applicable to controlling invasive Cane Toads in Japan.

## Introduction

Invasive species can have major ecological impacts on native taxa, and such impacts can be especially severe on islands that are refuges for endemic fauna [1,2]. Thus, managers need effective methods with which to reduce the abundance of pest species. Eradication is often difficult or impossible, but pro-active management can reduce local densities of the invasive species to levels that mitigate their environmental impacts [3].

One of the most-intensively-studied invaders worldwide is the Cane Toad (*Rhinella marina*), a large anuran that was introduced to more than 40 countries worldwide in ill-advised attempts at biocontrol of pests [4]. Extensive research has explored a wide range of potential methods to reduce toad numbers, ranging from genetically-engineered viruses [5] to larval-suppressant pheromones [6,7]. Realistically, the only feasible approach to control toad numbers will be multi-pronged, and include removal of adults as well as a reduction in recruitment [8]. Collecting and removing adult toads alone will have little impact [9,10]; but if combined with other methods for aquatic life-history stages (egg, embryo, and tadpole), may have substantial impacts on toad abundance [11].

**Fig 1:**
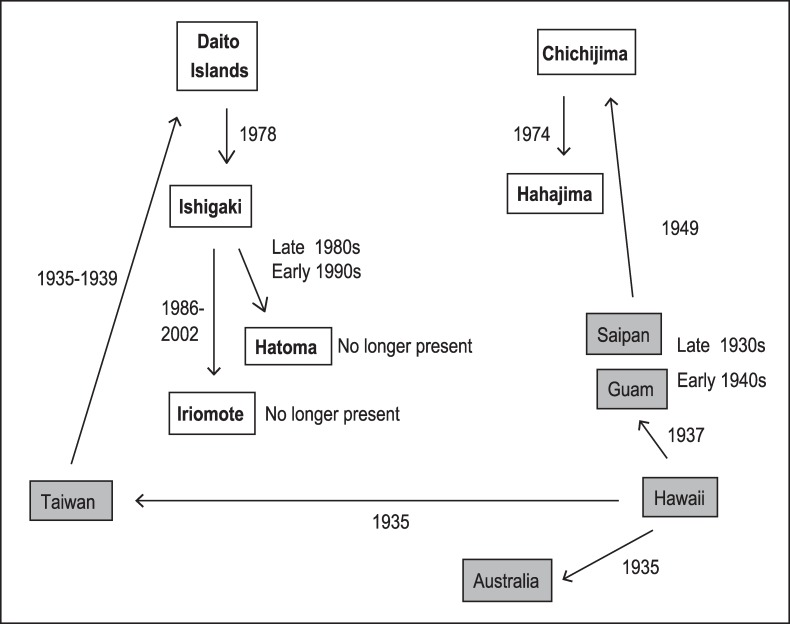
History of introductions of Cane Toads (*Rhinella marina*) to Japanese islands (in bold). In Iriomote, Cane Toads were transported from Ishigaki as accidental entries. Based on [4,21–23].

Despite the wide geographic spread of Cane Toad introductions, research on Cane Toad impact and control has been strongly focused in Australia (e.g. [11,12]). The Australian research has documented strong geographic variation in toad behaviour, reflecting rapid adaptation to Australian conditions as well as direct evolutionary effects of the invasion process [13,14]. Thus, for example, toads at the invasion front in Western Australia differ substantially in morphology, behaviour, endurance and immune function from conspecifics in long-colonised areas of eastern Australia [15–17], and many of these traits are heritable. [14,15,18,19]. Hence, control methods that work well with one population of Cane Toads may be less effective in other populations. Geographic divergence is especially likely if a newly-established population is founded by a small number of colonists, without gene flow from other areas, and is exposed to biotic and abiotic conditions different from those experienced by the ancestral stock [20]. Cane Toads in Japan fulfil those criteria (Fig 1). To control Cane Toads in areas other than Australia, then, we need to evaluate whether the methods developed in Australia can be applied to invasive populations in other parts of the world.

In this paper, we report experiments to test the applicability of three methods that have been developed from Australian research. The first is the discovery that chemicals produced by Cane Toad tadpoles reduce the viability of any conspecific embryos that encounter those chemicals [6,7,24]. Thus, those chemicals might offer a way to reduce recruitment rates of toads. The second method centres on the discovery that Cane Toad tadpoles are attracted to toxins produced by adult toads, such that funnel-traps baited with toad toxins can remove most or all toad tadpoles from natural waterbodies [25,26]. The third method involves the use of synthetic toad calls to attract adult Cane Toads to traps. In anurans, advertisement calls by males can elicit approaches by conspecifics [27], suggesting that we can control toads with acoustic traps [28]. The advertisement calls of larger male anurans have lower frequencies, and hence may be more attractive to females [29–32]. Like many other aspects of Cane Toad biology, however, call structure shows geographic variation [33], so that acoustic lures may need to be fine-tuned to local conditions.

## Materials and methods

### Ethics statement

Because eggs and tadpoles used in this study were not collected in a protected area, no permit was required from the relevant wildlife regulatory agency. The owners of the paddy fields from which tadpoles were collected gave permission for us to collect those specimens. Collection of eggs and tadpoles was done using small mesh nets, in accordance with the field research guideline of the Japan Ethological Society. The number of animals used was restricted to the minimum needed to achieve statistically robust comparisons. The study animal (*Rhinella marina*) is a pest species. None of the other three anuran species (*Microhyla ornata*, *Fejervarya sakishimensis*, *Rhacophorus owstoni*) are endangered or protected. All animal care procedures were authorised by the Ministry of the Environment of Japan (14000279), and experiments were conducted under the regulation of Kyoto University Ethics Committee.

### Study species

Cane Toads are among the largest anurans, with maximum body mass sometimes exceeding 1 kg [34,35]. However, adults generally average around 100 to 300 g, with females growing larger than males [4,13]. On Ishigaki Island in southern Japan, mean body sizes in our sample of collected Cane Toads averaged 116.82 g (106.02 mm snout-urostyle length [SUL]) in males, and 168.49 g (113.82 mm SUL) in females (comparing the sexes, *t*-test, SUL: t = 2.71, P < 0.01, mass: t = 5.54, P < 0.0001).

### History of translocation

Cane Toads are native to parts of Central and South America, but were moved to many other places around the world to control insect pests. In 1932, 150 Cane Toads were translocated from Puerto Rico to Hawai’i [4]. In 1935, descendants of these toads were taken from Hawai’i to Taiwan; and over the next four years that Taiwanese population was the source of further translocations to the Daito Islands in Japan (see Fig 1, [4]). In 1978, approximately 10–15 toads were moved from the Daito Islands to Ishigaki, in southern Japan [36]. Ishigaki is one of the southernmost islands within an archipelago that stretches southwest from Okinawa (24°36´52´´N, 124°15´62´´E), and the island has an area of 223 km^2^. Cane Toads are found throughout the island, with the total population likely to exceed 20,000 individuals (H Ota, personal communication). Ishigaki experiences a subtropical climate, and Cane Toads breed there in a wide range of waterbodies such as rice paddy fields, ponds, streams, and artificial pools in sugarcane fields. These waterbodies are found throughout the island. In total, freshwater bodies that could be used by Cane Toads as breeding sites on Ishigaki comprise four large dams / lakes (total area 1.6 km^2^), agricultural land that contains extensive irrigation systems (total area 70.5 km^2^; 32% of island land mass) and numerous streams running from mountainous areas to the ocean. Area estimates were made using Google Earth Pro. Habitat and breeding areas on Ishigaki such as sugarcane fields are similar those used by Cane Toads in coastal regions of eastern Australia, but different from more arid areas of northern, western and inland Australia where Cane Toads also occur.

### Need for control

On Ishigaki, Cane Toads have caused the death of native snakes that are fatally poisoned when they attempt to consume the toads [37]. Although data are limited from Japan, the same mechanism of impact has devastated populations of some large predator species in Australia [12]. Accordingly, Cane Toads represent a potential problem for endemic wildlife on Ishigaki.

### Collection of animals

Adult toads and tadpoles were hand-collected from sugarcane and rice paddy fields; adults (318 individuals) were collected from southern, central and northern sections of Ishigaki island, while tadpoles (200 individuals) were collected from the southern section. Eggs of native anurans (Owston’s Green Tree Frog *Rhacophorus owstoni*, Sakishima Rice Frog *Fejervarya sakishimensis*, Ornate Narrow Mouthed Frog *Microhyla ornata*) were also collected from southern and central locations (two egg clutches per species). Cane Toad eggs were obtained by injecting adult toads subcutaneously with 0.25 mg/ml leuprorelin acetate in the laboratory. Males were injected with 0.25 ml and females 0.75 ml, and left overnight as pairs to spawn in 80-litre tubs with water. Fourteen pairs of toads were injected, of which six pairs laid eggs. Eggs of all species developed into tadpoles in the laboratory, and were then kept in 100 L plastic containers on a diet of algae pellets (Hikari Algae Wagers, Kyorin, Japan) fed ad libitum until used in experiments.

### Embryonic suppression experiments

We conducted a series of experiments to investigate (1) sensitivity of Cane Toad embryos to suppression by toad tadpoles, (2) sensitivity of Cane Toad embryos to suppression by frog tadpoles, and (3) sensitivity of frog embryos to suppression by Cane Toad tadpoles and frog tadpoles. For (1), we exposed embryos from four Cane Toad egg clutches to chemical cues of four groups of conspecific tadpoles (two laboratory-bred tadpole sibships, and two groups of wild tadpoles each collected from a single school that may, or may not, have been sibships). Each of the four egg clutches was exposed to cues from two or three of the tadpole groups. For (2), we exposed Cane Toad embryos from two egg clutches to tadpole chemical cues of three native anurans (*F*. *sakishimensis*, *R*. *owstoni*, *M*. *ornata*; one sibship each). For (3), we exposed embryos from one clutch of *M*. *ornata* to chemical cues of two wild-collected groups of Cane Toad tadpoles, and one sibship each of native tadpoles *F*. *sakishimensis* and *M*. *ornata*.

Experiments were conducted in the laboratory during May 2015 using 1-litre plastic containers filled with 750 ml water. Containers were divided in half using fibreglass mesh (1mm mesh size). Ten embryos (Gosner stage 17–18 [38]) were placed on one side of the mesh barrier, while either zero (Control) or three (Treatment) tadpoles were placed on the other side (N = 5 replicates per treatment). Treatments were randomly assigned to containers, and eggs / tadpoles were randomly assigned to treatments.

When embryos had developed into mobile, feeding tadpoles (stage 25; approximately 72 hours) we counted the number surviving in each container, and then removed the stage 25 tadpoles from exposure to older tadpole cues. For each container, five of these stage 25 tadpoles were haphazardly chosen and transferred to new 1-litre containers filled with 750 ml water. These tadpoles were allowed to grow for 10 days, being fed to excess (Hikari Algae Wafers, Kyorin, Japan) and with daily water changes. After 10 days, we counted the number of surviving tadpoles in each container, and recorded tadpole body mass (to 0.001 g) and developmental stage [38].

### Tadpole attraction experiments

Experiments were conducted in the laboratory during May 2015 using plastic trays (53 cm × 32 cm × 6 cm) filled with 12 litres of water. We tested attraction responses by Cane Toad tadpoles to two cues: (1) water that had previously contained Cane Toad eggs, and (2) toxin from adult Cane Toads. Egg water cues were generated by placing 30 cm sections of egg string (in total, 3–4 m, Gosner stage 11–19) from two egg clutches into water for six hours, and then egg water was frozen until used the following day. Adult toad toxin was obtained by squeezing 100 mg toxin (total mass) directly from the parotoid glands of three adult Cane Toads into 75 ml plastic cups containing 30 ml water, and allowed to stand for 1 hour. Each cue was tested in a separate experiment.

We placed groups of 20 Cane Toad tadpoles from a mixture of four tadpole groups (see above for details) in each of 8 trays and allowed them to acclimate for 60 min. After this, we placed a 1-litre plastic container with a single funnel entrance (external diameter = 40 mm, internal diameter = 10 mm) in a corner of each tray. Traps were baited with either a 30 ml solution of egg water (thawed to room temperature) or toxin (as described above). Control traps received 30 ml water (N = 4 replicates per treatment). Treatments were randomly assigned to trays, and tadpoles were randomly assigned to treatments. We observed tadpole swimming behaviour throughout the experiments, specifically looking for the commencement of frenetic feeding responses, and counted the number of tadpoles in traps every 5 minutes for 30 minutes, and then every 10 minutes for a further 60 minutes (90 minutes total experiment time).

### Acoustic experiments

We recorded advertisement calls of one male Cane Toad in the field on Ishigaki using an IC-recorder (Olympus, LS-14) and microphone (Olympus, ME31). This was used as the base call, and had a dominant frequency of 657 Hz (analyzed using Adobe Audition CS6 [version 5.0]). The recording was used to generate acoustic treatments 2, 4 and 5 below. We also recorded a chorus of five to eight male Cane Toads for acoustic treatment 3 below. This group chorus included low and high frequency calls (591–715 Hz).

We exposed both male and female adult toads (159 males, 145 females) to five sound types, as follows: (1) pink noise (control: Audio Check CD: DENON, Nippon Columbia Co., Ltd.); (2) single male toad call; (3) group chorus of five to eight male toads; (4) high-frequency male toad call (the artificial call of a single male toad manipulated [in Pro Tools LE] to raise the dominant frequency from 657 to 734 Hz); and (5) low-frequency male toad call (the artificial call of a single male toad manipulated [in Pro Tools LE] to reduce the dominant frequency from 657 to 564 Hz).

Playback experiments were conducted between February 2014 and December 2015 in the field on Ishigaki, and followed the methods described by Schwarzkopf and Alford in Australia [28]. Trials were conducted in a 7m-diameter circular arena constructed of wire netting (60 cm high), with red clay as the substrate. In this experiment, a single toad was exposed to one of five sound treatments (listed above; treatment selected randomly) at a volume of approximately 50dB at 3.5 m (from the center of circle to the wire netting wall). Bricks were placed at 30° intervals around the arena, and a speaker (Elecom, LBT- SPP20BK) was placed at the side of one of these bricks to camouflage the speaker. The toad was placed in the center of the arena at the start of each trial. The observer switched on the IC recorder (Olympus, LS-14), and retreated behind cover to be unseen by the toad. After ten minutes, the observer illuminated the arena with a spotlight, and recorded the location of the toad in the arena (compass direction of the toad from the center, and distance from the speaker). Toads tended to turn away from the light when they were illuminated [as per 28], so we did not record the direction they were facing. Each toad was used in only one trial, and all individuals were used in experiments on the same night that they were captured.

### Statistical analysis

#### Embryonic suppression

Survival of embryos to tadpole stage 25 (i.e. prior to the commencement of the 10 day growth period) was >98% for all anuran species in all treatments. Therefore, we did not formally analyse survival effects for these early embryonic stages. For the 10 day growth period post stage 25, we analysed survival effects for Cane Toad tadpoles that had been exposed to conspecific tadpole cues during embryonic development (proportion of tadpoles alive / dead per treatment at day 10) using logistic regression [39], incorporating Egg Clutch as a random factor. Analysis was performed in R [40] based on the quasi-binomial distribution to account for over-dispersion of data. We did not formally analyse survival effects for toad eggs exposed to frog tadpoles, or frog eggs exposed to toad tadpoles / frog tadpoles, due to the high survival rates in these experiments (>95% survival).

For experiments testing responses of toad eggs to cues from conspecific tadpoles and native frog tadpoles (multiple toad egg clutches per experiment), we analysed effects on growth and development at day 10 using ANOVA with Egg Clutch as a random factor and Treatment (Control / Tadpole Group) as a fixed effect. To assess responses of *M*. *ornata* eggs (one egg clutch) to cues from Cane Toad tadpoles and native frog tadpoles, we used Dunnett’s tests to compare growth and development responses relative to the Control Treatment. All growth and development analyses used container mean data to avoid pseudo-replication. Mass data were log-transformed prior to analysis to satisfy all model assumptions were met, including normal distribution of residuals. Analyses were performed using JMP (Version 9.0.0, SAS Institute Inc., Cary, NC, 1989–2007; [41]).

#### Tadpole attraction to toad egg water and adult toad toxin

We compared the proportion of Cane Toad tadpoles in control traps versus traps containing conspecific egg water or conspecific adult toxin using logistic regression, with Time as a random factor. Analyses were performed in R based on the quasi-binomial distribution to account for over-dispersion of data.

#### Adult attraction to acoustic cues

We normalised the position of the speaker to zero, and used Rayleigh’s test to assess whether the angles of movement selected by the toads departed significantly from random [42]. To determine whether any of the sound treatments were better than the control for attraction, we compared the distance of the toad from the speaker in the Control treatment to the distance from the speaker in all other sound treatments using Dunnett’s tests. Statistical analyses were performed using Oriana 4 and JMP 9.0 [41,43].

Data from this study have been deposited in Dryad (10.5061/dryad.1mt16).

## Results

### Embryonic suppression

**Fig 2:**
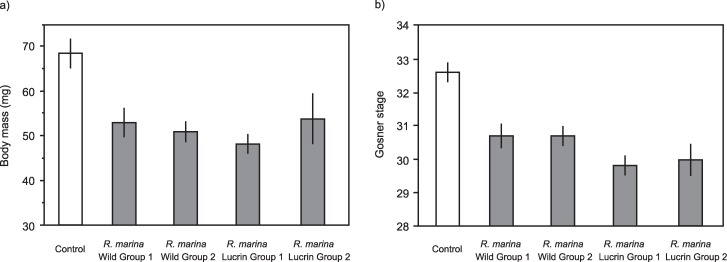
Growth and development of Cane Toad tadpoles at day 10 following exposure as embryos to chemical cues of older conspecific tadpoles. The panels show impact of exposure to older tadpoles on a) body mass, and b) developmental stage (Gosner 1960 [38]) of embryos that were exposed to cues from tadpole of four groups (Wild Group 1, 2, and Lucrin Group1, 2). The graphs show mean values with associated standard errors.

After 10 days, survival of Cane Toad tadpoles in the Control treatment was 91 +/- 5% SE, while survival of tadpoles exposed as embryos to the four groups of conspecific older tadpoles ranged from 80% to 100% (Lucrin Group 1: 80 +/- 12% SE, Lucrin Group 2: 88 +/- 8% SE, Wild Group 1: 89 +/- 11% SE, Wild Group 2: 100 +/- 0% SE). These survival rates were not significantly different from the Control (p = 0.40, 0.84, 0.91 and 0.99, respectively). However, toad eggs exposed to chemical cues from conspecific tadpoles subsequently exhibited reduced growth (body mass: F = 8.50, df = 4,4, p < 0.0001; Fig 2a) and development (Gosner stage: F = 12.25, df = 4,4, p < 0.001; Fig 2b). Tukey’s HSD tests showed that, for both growth and development, the magnitudes of suppression induced by each of the four older tadpole groups were indistinguishable from each other, and all had a greater negative effect than the Control treatment.

**Fig 3:**
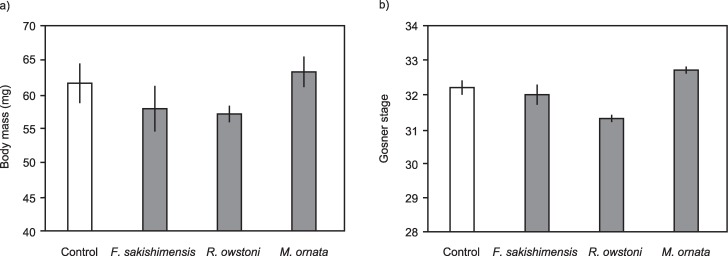
Growth and development of Cane Toad tadpoles at day 10 day following exposure as embryos to chemical cues of older tadpoles of native frog species (*F*. *sakishimensis*, *R*. *owstoni*, *and M*. *ornata*). The panels show impact of exposure to native frog tadpoles on a) body mass, and b) developmental stage (Gosner 1960 [38]) of Cane Toad embryos. The graphs show mean values with associated standard errors.

Exposure to native frog tadpoles (*F*. *sakishimensis*, *R*. *owstoni*, *M*. *ornata*) did not significantly affect Cane Toad eggs in terms of growth (F = 2.27, df = 3, 3, p = 0.10) or development (F = 2.31, df = 3, 3, p = 0.10; Fig 3).

**Fig 4:**
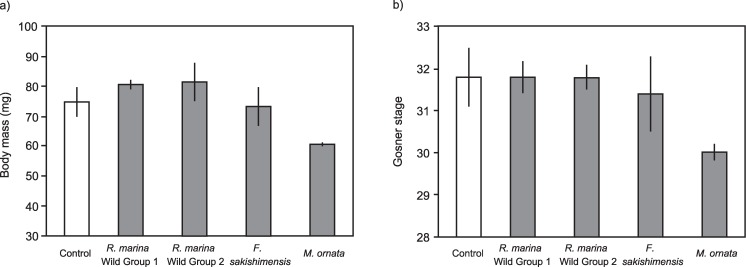
Growth and development of tadpoles of the Ornate Narrow Mouthed Frog *M*. *ornata* at day 10 day following exposure as embryos to chemical cues of older Cane Toad tadpoles (Wild Group 1 and 2) and native frog tadpoles (*F*. *sakishimensis and M*. *ornata*). The panels show impact of exposure to tadpoles on a) body mass, and b) developmental stage (Gosner 1960 [38]) of Ornate Narrow Mouthed Frog embryos. The graphs show mean values with associated standard errors.

Exposure of *M*. *ornata* eggs to chemical cues from Cane Toad tadpoles had no significant effect on subsequent growth (Wild Group 1 tadpoles p = 0.33, Wild Group 2 tadpoles p = 0.30; Fig 4a) or development (Wild Group 1 tadpoles p = 1.00, Wild Group 2 tadpoles p = 1.00, Fig 4b). Similarly, exposure of *M*. *ornata* eggs to cues from native frog tadpoles had no significant effect on subsequent growth (*F*. *sakishimensis* tadpoles p = 1.00, *M*. *ornata* tadpoles p = 0.18; Fig 4a) or development (*F*. *sakishimensis* tadpoles p = 0.94, *M*. *ornata* tadpoles p = 0.09; Fig 4b), despite a trend for reduced growth and development following exposure to conspecific tadpoles.

### Tadpole attraction

**Fig 5:**
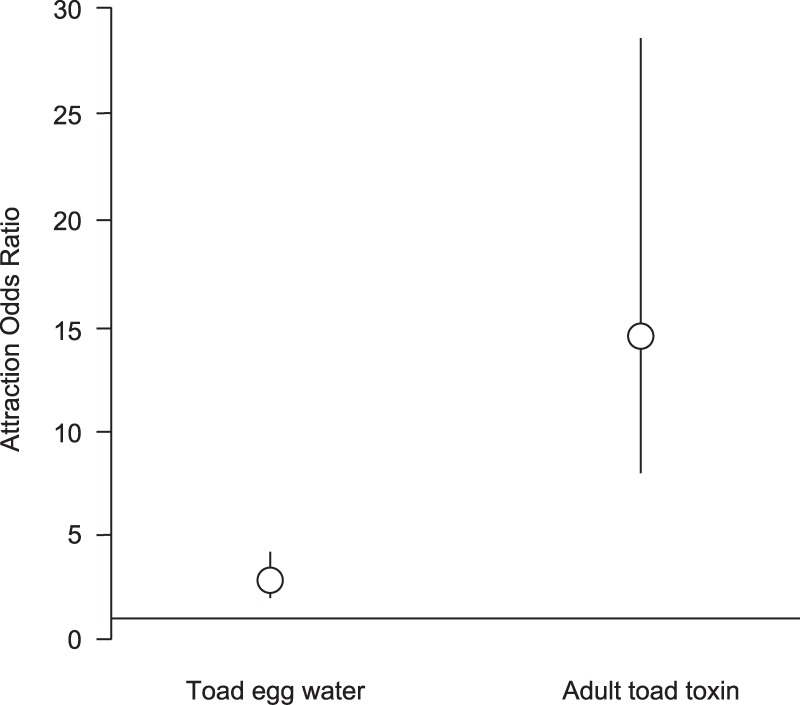
Odds ratio for attraction responses of Cane Toad tadpoles to cues from conspecific eggs and adult toxin. The graph shows mean odds ratios with 95% confidence intervals. The horizontal line at Odds Ratio of 1.0 represents the Control; treatments whose 95% confidence intervals do not overlap this line are significantly different from the Control.

Cane Toad tadpoles showed strong responses to traps containing conspecific egg water or adult toxin relative to Controls, Within 15 minutes, >50% of tadpoles in all replicates of egg water and adult toxin treatments showed frenetic swimming activity (= feeding activity) at the entrance of the trap funnels. In contrast, no tadpoles in the Control replicates were observed to show such behaviour. After 90 minutes, tadpoles showed significant attraction to both conspecific egg water and adult toxin (in both instances p < 0.001). On average, toad tadpoles were 2.9 times more likely to enter traps containing toad egg water, and 14.5 times more likely to enter traps containing adult toxin, relative to Control traps (Fig 5).

### Attraction of adult toads to acoustic cues

**Fig 6:**
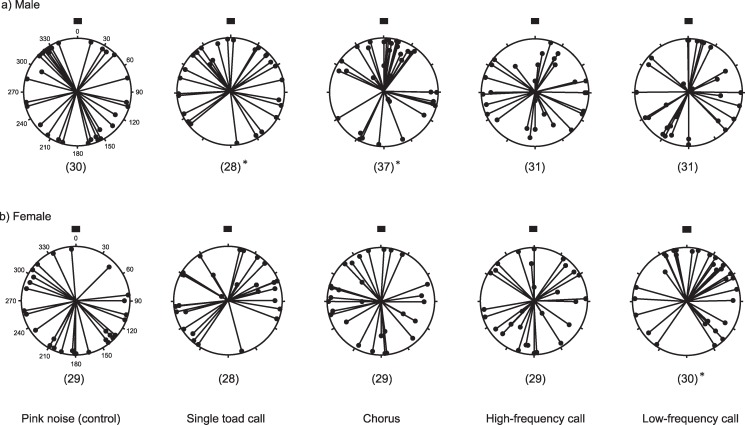
Angle that Cane Toads approached a variety of acoustic stimuli after 10 minutes. The panels show responses of (a) male toads (upper panel), and (b) female toads (lower panel). Sample size is given in parentheses. The black square indicates the position of the speaker. Each black small circle shows the position of a toad after ten minutes. * P<0.05.

**Fig 7:**
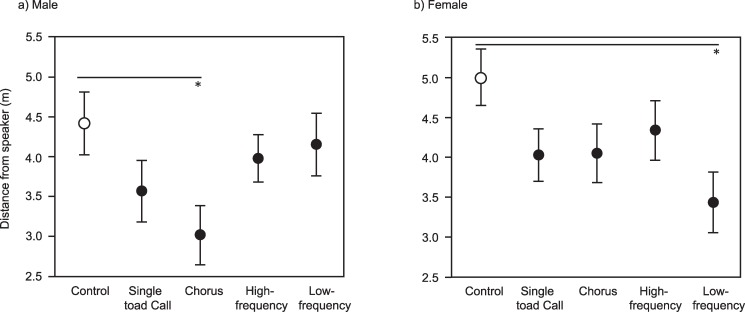
Phonotactic responses of Cane Toads (*Rhinella marina*) to a variety of acoustic stimuli, based on the toad’s distance to the speaker after 10 minutes. The panels show responses of (a) male toads, and (b) female toads. All graphs show mean values and associated standard errors. * P<0.05.

Tables 1 and 2 show the results of statistical analyses of toad position (angles of movement and distance from speaker) after ten minutes for male and female Cane Toads, respectively. Males were significantly non-randomly dispersed within the arena in the treatments that consisted of both the single toad call (Rayleigh’s V = 2.186, P = 0.014) and the chorus (Rayleigh’s V = 3.039, P = 0.001, Table 1, Fig 6a). Female toads were non-randomly dispersed towards the low-frequency call (Rayleigh’s V = 2.158, P = 0.015, Table 2, Fig 6b). Relative to the control, male toads were significantly attracted to the male chorus (Dunnett’s test P = 0.024, Table 1, Fig 7a) whereas female toads were attracted to the single male toad low-frequency call (Dunnett’s test P = 0.009, Table 2, Fig 7b).

**Table 1:** Results of statistical analysis on data for position within the acoustic-testing apparatus for male Cane Toads after 10 minutes. We used Rayleigh’s tests to assess the directional mean of toads, and Dunnett’s tests to compare the distance from speaker in the pink noise (Control) treatment versus the other four sound types.

Sound Type	N	Rayleigh’s Test (V)	Rayleigh’s Test (p)	Dunnett’sTest (p)
Pink Noise (Control)	31	-0.068	0.527	
Single Toad Call	28	2.186	0.014	0.338
Chorus	38	3.039	0.001	0.024
High-Frequency Call	31	0.239	0.406	0.827
Low-Frequency Call	31	-0.627	0.734	0.965

**Table 2:** Results of statistical analysis of data for position within the acoustic-testing apparatus for female Cane Toads after 10 minutes. Statistical methods were same as described in the caption to Table 1.

Sound Type	N	Rayleigh’s Test (V)	Rayleigh’s Test (p)	Dunnett’s Test (p)
Pink Noise (Control)	29	-1.724	0.958	
Single Toad Call	28	1.168	0.122	0.187
Chorus	29	-0.156	0.562	0.199
High-Frequency Call	29	-0.604	0.726	0.498
Low-Frequency Call	30	2.158	0.015	0.009

## Discussion

Our experiments generated an encouraging result: the intraspecific growth, developmental and behavioural responses recently identified for potential control of Cane Toads in Australia were also observed on Ishigaki Island in southern Japan. Cane Toads in Australia and Japan have been genetically separated for at least 80 years, and have gone through successive translocation events that presumably resulted in significant genetic bottlenecks (Fig 1, [20]). Nonetheless, the responses we recorded in Japan were very similar to those reported for Australian toads.

In our study of Cane Toads in Japan, mean effect size (Cohen’s *d*) for suppression of growth following embryonic exposure to conspecific tadpoles was -1.0 (relative to the Control; negative effect size indicates reduced growth), which is comparable to a suppression growth effect size of -0.9 in Australia (Australian data re-worked from [24]). In addition, tadpole attraction responses to both cue types were similar between countries. In Japan, Cane Toad tadpoles were, on average, 2.9 times more likely to enter traps baited with toad egg water than Control traps, versus 5.2 times more likely in Australia (Australian data reworked from [25]). However, the mean estimate for responses in Japan of 2.9 lies within the 95% CI intervals for responses in Australia (CI = 2.7–10.4), indicating a statistically non-significant difference between countries. For responses to traps baited with adult toxin, Cane Toad tadpoles in Japan were on average14.5 times more likely to enter treatment traps than Control traps, versus 17.0 times more likely in Australia (Samantha McCann unpublished data, corrected for equivalent mass of toxin used as bait). The mean estimate of 17.0 in Australia lies with the 95% CI intervals for Japan (CI = 8.1–28.6), again indicating statistical non-significance between countries. We did not explicitly compare effect sizes for responses of adult Cane Toads to acoustic cues because (1) tests in the two countries used different cues (primarily single male calls in Japan versus male chorus calls in Australia), and (2) the results provided in (28) are not sufficient to calculate an effect size for toad responses in Australia. Nonetheless, the above assessment demonstrates the quantitative comparability of embryonic and larval Cane Toad responses in Japan and Australia.

Cane Toad embryos in Japan were sensitive to chemical cues from older conspecific tadpoles, with exposure to these cues reducing subsequent rates of growth and development. This response appears to be species-specific on Ishigaki: Cane Toad embryos responded to cues from conspecific tadpoles but not from native frog tadpoles, whereas frog embryos did not respond to cues from toad tadpoles. Similar specificity has also been demonstrated in Australia, where native frog embryos are unaffected by exposure to Cane Toad tadpoles [7], and toad embryos are unaffected by exposure to native frog tadpoles (G. Clarke unpublished data). Further work (increasing the sample size for native frog eggs and tadpoles, including testing other native species on Ishikgai Island) is required to confirm our initial results on Ishigaki, but the specificity demonstrated to date is promising for targeted control of Cane Toads in the embryonic stage.

Cane Toad tadpoles in Japan also showed strong behavioural responses to cues from conspecific eggs and adult toxin. Again, this mirrors results in Australia [25,26], demonstrating a similarity in behavioural ecology within the species in separate invasive populations. Cane Toad tadpoles are highly cannibalistic both in Australia and Japan ([44], Haramura unpublished data) and use the cues from developing conspecific eggs to locate and consume them [25]. Toxin from adult Cane Toads also induces an attraction response. In Australia, this response is species-specific, with native frog tadpoles showing no evidence of attraction, and in fact being repulsed by adult toad toxin [26]. Attraction specificity in Japan is currently unknown and needs to be further investigated (i.e. whether or not tadpoles of native species on Ishigaki Island are attracted to Cane Toad toxin), but the specificity for suppression (see above) is promising in terms of species-specificity for attraction responses also. Additionally, no native species of Bufonidae occur on Ishigaki or indeed on any of the other Japanese islands where Cane Toads currently exist (Ogasawara, Minami-kita Daito-Jima), further reducing the likelihood of collateral damage for native anuran larvae even if larval suppression / attraction pheromone effects occur across Bufonid species (a hypothesis not yet tested).

To design a program to trap Cane Toad tadpoles, a fundamental question is: what is the most effective bait to use? In Japan, attraction of Cane Toad tadpoles to adult toad toxin was five times greater than attraction to egg water (compared to 3 times greater in Australia). This difference in attraction may be due to differences in the quantity and/or composition of attraction chemicals in different life-history stages: adult toxin may contain greater concentrations of attractant chemicals, or different chemicals that induce a greater attraction response, than toad eggs. Understanding and utilising such differences would be important to maximise the effectiveness of future efforts to trap toad tadpoles. Another factor worth considering is whether attractant chemicals vary in identity between countries. Would Cane Toad tadpoles in Japan show even greater attractant response if exposed to chemicals derived from eggs or adult toxin of Cane Toads in Australia (or vice versa)? Again, understanding such differences would contribute to more effective removal of Cane Toad tadpoles in invasive populations.

In our playback experiments, adult Cane Toads of both sexes moved towards the acoustic stimuli of male toad calls, but the optimal stimulus differed between sexes (males were attracted to the chorus call, and females to the low-frequency call). Research in Australia reported that both male and female Cane Toads are attracted to quiet (47 dB(A) at 1 m) recordings of a small toad chorus, whereas only male toads are attracted to loud recordings (67 dB(A) at 1 m), suggesting that this sex-specific response could be useful for controlling populations of this highly invasive species [28]. Our acoustic results are similar to those from Australia (at least in broad terms), suggesting that traps that include acoustic attractants would be effective in capturing invasive Cane Toads (and perhaps, targeting female toads) in Japan also. Removal of female toads is considered to be the most effective way of reducing the overall toad population in the long term [8,11] because each female Cane Toad can lay more than 30,000 eggs. Nonetheless, removing both sexes would have a more immediate effect on current population size, and having the capacity to attract both male and female Cane Toads using acoustic traps provides maximum flexibility for any control program.

Acoustic signals play an important role in intraspecific communication for most anurans. Male anurans use the calls of others to locate waterbodies for hydration or mating opportunities [45–50]. These sounds can propagate over several hundred meters to provide a long-range cue [51]. Increased chorus activity is associated with increasing mating probabilities for males [27, 52, 53], which may explain why male Cane Toads in our experiments were more strongly attracted to chorus signals than to any of the other advertisement calls that we played.

Female anurans also use acoustic cues to locate waterbodies for hydration, and to select males for breeding [54,55]. Many female anurans prefer call parameters that are related to morphological characteristics of the caller [56]. Larger males have lower call frequency, and females exhibit a strong preference for low frequency calls [57,58]. In keeping with this pattern, female Cane Toads showed a strong tendency to choose calls with a low dominant frequency, which may indicate a preference for large males [59–62]. In the present study, the synthetic low-frequency call was designed to mimic the call of an unusually large male toad. Why, then, were females not attracted to the chorus call, which included low-frequency as well as high-frequency calls? Persistent courtship can be costly for female Cane Toads [63], and female anurans in amplexus sometimes leave areas of high male density to escape from intense harassment [64,65]. In the present study, the chorus call may not attract females because a large calling aggregation may impose unacceptably high costs of harassment for females. It is also worth noting that we only manipulated the frequency of the advertisement call of a single male Cane Toad. Future studies could usefully explore the impacts of varying pulse frequency, call rate, and pulse number, as well as dominant frequency of the call, all of which are known to influence mate choice in female anurans [53, 56, 66, 67].

Our experiments, although preliminary, are encouraging for the feasibility of using pheromonal and acoustic cues to control invasive populations of Cane Toads in Japan. In addition, the fact that these cues occur in Australia and Japan is encouraging for the potential to control populations of Cane Toads in other countries where the species has been introduced [4].
